# Real-world characteristics of “super-responders” to mepolizumab and benralizumab in severe eosinophilic asthma and eosinophilic granulomatosis with polyangiitis

**DOI:** 10.1183/23120541.00419-2023

**Published:** 2023-10-30

**Authors:** Andrea Portacci, Raffaele Campisi, Enrico Buonamico, Santi Nolasco, Corrado Pelaia, Nunzio Crimi, Alida Benfante, Massimo Triggiani, Giuseppe Spadaro, Maria Filomena Caiaffa, Giulia Scioscia, Aikaterini Detoraki, Giuseppe Valenti, Francesco Papia, Alessandra Tomasello, Nicola Scichilone, Girolamo Pelaia, Claudia Crimi, Giovanna Elisiana Carpagnano

**Affiliations:** 1 Institute of Respiratory Disease, Department of Translational Biomedicine and Neuroscience, University “Aldo Moro”, Bari, Italy; 2 Respiratory Medicine Unit, Policlinico “G. Rodolico-San Marco” University Hospital, Catania, Italy; 3 Department of Clinical and Experimental Medicine, University of Catania, Catania, Italy; 4 Department of Health Sciences, University “Magna Graecia” of Catanzaro, Catanzaro, Italy; 5 Division of Respiratory Diseases, Department of Health Promotion Sciences, Maternal and Infant Care, Internal Medicine and Medical Specialties (PROMISE), University of Palermo, Palermo, Italy; 6 Division of Allergy and Clinical Immunology, University of Salerno, Salerno, Italy; 7 Center for Basic and Clinical Immunology Research (CISI), University of Naples Federico II, Naples, Italy; 8 Department of Medical and Surgical Sciences, School and Chair of Allergology and Clinical Immunology, University of Foggia, Foggia, Italy; 9 Department of Medical and Surgical Sciences, University of Foggia, Foggia, Italy; 10 Division of Internal Medicine and Clinical Immunology, Department of Internal Medicine and Clinical Complexity, Azienda Ospedaliera Universitaria Federico II, Naples, Italy; 11 Allergology and Pulmonology Unit, Provincial Outpatient Center of Palermo, Palermo, Italy; 12 These authors contributed equally

## Abstract

**Background:**

The current definition of severe eosinophilic asthma (SEA) super-responders to biologic treatment does not include patients with other eosinophil-based comorbidities. Although eosinophilic granulomatosis with polyangiitis (EGPA) is frequently associated with SEA, we lack data on a possible super-response to biologic treatments in patients suffering from these two diseases. We aim to assess super-responder features in real-life patients with SEA and EGPA treated with mepolizumab and benralizumab.

**Methods:**

We enrolled 39 patients with SEA and EGPA eligible for treatment with mepolizumab or benralizumab. Super-responder assessment was performed considering oral corticosteroid (OCS) cessation, lack of exacerbations, forced expiratory volume in 1 s and Asthma Control Test (ACT) improvement.

**Results:**

Super-responders showed worse clinical baseline characteristics than non-super-responder patients, with a greater improvement in severe asthma exacerbations, OCS dose reduction and ACT score increase. Definition of super-responders was consistent only considering a 12-month course of monoclonal antibody, lacking sensitivity in earlier evaluations.

**Conclusion:**

Mepolizumab and benralizumab are safe and effective in patients with EGPA and SEA, since a consistent proportion of patients show a super-response after 12 months of treatment. Further studies will address specific criteria for super-responder assessment in these patients.

## Tweetable abstract

**Patients suffering from severe asthma and EGPA frequently show a super-response to anti-IL-5/IL-5R biologics, despite showing worse baseline clinical features, such as more severe exacerbations and more frequent use of systemic corticosteroids** https://bit.ly/45DCRZi

## Introduction

Eosinophilic granulomatosis with polyangiitis (EGPA) is a systemic disease characterised by a granulomatous vasculitis affecting small vessels, severe asthma and sinus disease. Similar to severe eosinophilic asthma (SEA), the natural history of EGPA is deeply influenced by eosinophilic inflammation, which is one of the main pathophysiologic drivers of this disease [1]. Consequently, several authors proposed anti-interleukin (IL)-5/IL-5-receptor monoclonal antibodies as valid treatment options in patients with simultaneous EGPA and SEA [2–4] and a randomised trial performing a head-to-head comparison between the two anti-IL-5 biologics is still ongoing (MANDARA, clinicaltrials.gov identifier NCT04157348). However, clinical response to monoclonal antibodies can be heterogeneous, challenging clinicians to define which cluster of patients has the greatest improvement after a course of treatment. As a matter of fact, SEA “super-responders” have been variously defined according to multiple clinical, functional and biological features [5]. According to current literature, there are two generally accepted criteria to define a super-responder: the lack of asthma exacerbations and the discontinuation of oral corticosteroids (OCS) after ≥12 months of biologic treatment [6, 7]. Upham *et al.* [8] defined super-responders using a Delphi-based survey of experts, combining several major and minor criteria such as the assessment of asthma control improvement, forced expiratory volume in 1 s (FEV_1_) increase and, as mentioned earlier, OCS cessation and the absence of exacerbations after a 1-year course of monoclonal antibody administration. Similarly, Kavanagh and co-workers [6, 7] transposed these concepts into real-life evidence, classifying super-responders strictly according to the absence of OCS use or exacerbations after 24 and 48 weeks of treatment with mepolizumab [7] and benralizumab [6].

Nevertheless, the available definitions of super-responders were developed only considering patients with SEA and overlooking other eosinophil-driven comorbidities. It is well known that patients with EGPA frequently develop SEA [9], but whether SEA super-responder classification could still be reliable in patients with EGPA is still to be clarified.

The aim of our study is to verify the suitability of severe asthma super-response definition in patients suffering from SEA and comorbid EGPA, exploring which features could predict a response to biologic treatments.

## Material and methods

### Study design

We conducted a retrospective, observational, multicentre analysis from the Southern Italy Network on Severe Asthma Therapy, including data on patients who underwent a 1-year course of mepolizumab or benralizumab from September 2017 to March 2022.

### Study population

Overall, from the 650 patients registered in our database, we screened 49 patients aged >18 years diagnosed with SEA and EGPA (supplementary figure E1). SEA diagnosis was made according to Global Initiative for Asthma (GINA) [10] and European Respiratory Society/American Thoracic Society [11] recommendations, while EGPA was assessed following 2022 American College of Rheumatology/European Alliance of Associations for Rheumatology classification criteria [12]. For each patient, the baseline evaluation (T0) included anthropometric and anamnestic data, comorbidity assessment, functional evaluation with flow–volume spirometry and blood eosinophil count (BEC). SEA clinical evaluation included Asthma Control Test (ACT), as well as information concerning the number and the severity of asthma acute exacerbations and the use of OCS. Patients receiving high-dose inhaled corticosteroids–long-acting β-agonists who remained uncontrolled despite the treatment were eligible for monoclonal antibody administration. Adherence to inhaled treatment was assessed using 12-items Test of Adherence to Inhalers (TAI). Monoclonal antibodies were prescribed only in presence of a TAI score ≥50 points, while patients with poor medication adherence were retrained and encouraged to use their inhaling devices. In case of poor treatment response despite a correct inhaled treatment course, clinicians could prescribe monoclonal antibodies according to specific criteria. Mepolizumab 100 mg was prescribed once a month in patients with BEC >150 cells·mm^−3^ (with a single value of blood eosinophils >300 cells·mm^−3^ in the past year) and ≥6 months of OCS treatment or two or more exacerbations (treated with OCS or hospitalisation) in the past 12 months. Benralizumab 30 mg was administered in patients with BEC ≥300 cells·mm^−3^ and an OCS treatment or two or more exacerbations (treated with OCS or hospitalisation) in the past year.

In order to assess SEA super-responders, we used a subset of major and minor criteria. Major criteria comprised 1) no exacerbations after 12 months of biologic treatment; 2) no OCS use after the start of monoclonal antibody administration; and 3) ACT improvement ≥6 points after 1 year of treatment. Minor criteria comprised 1) decrease in exacerbations ≥75% from baseline; 2) good symptom control (ACT ≥20 points); and 3) FEV_1_ improvement ≥500 mL from baseline.

SEA super-response was defined in presence of at least three features with two or more major criteria fulfilled. In case of complete absence of exacerbations, the first minor criterion (exacerbation decrease) was not considered for super-responder definition, avoiding overestimating treatment response.

To assess EGPA severity, we used the Birmingham Vasculitis Activity Score (BVAS), as well as anti-neutrophil cytoplasmic antibodies, to state the impact of vasculitis along with SEA. Moreover, EGPA pulmonary and extrapulmonary manifestations were also reported, as well as the use of any immunosuppressant drugs. EGPA relapses were defined as the presence of a worsening of EGPA clinical features (assessed with BVAS increase from baseline or with signs/symptoms of extrapulmonary organ damage) or with a worsening of asthma and/or ear–nose–throat manifestations leading to the increase in prednisone/prednisolone dose >4 mg·day^−1^, to the start of an immunosuppressive treatment or to a hospitalisation.

To define EGPA remission, we used two separate criteria, based on BVAS and OCS maintenance dose after 12 months of treatment with monoclonal antibodies: 1) “remission 1”: BVAS=0 and OCS dose ≤4 mg [13]; 2) “remission 2”: BVAS=0 and OCS dose ≤7.5 mg [14].

After T0 assessment, patients underwent follow-up visits after 1 (T1), 3 (T3), 6 (T6) and 12 (T12) months from the start of monoclonal antibody administration. Exclusion criteria were lack of treatment adherence, patients lost to follow-up, missing data on some main severe asthma features (OCS administration, exacerbations, symptoms) and patients who withdrew from the study.

The study was approved by the institutional ethics committees (ethics committee number 6313) and was conducted following the Helsinki Declaration of 1975 and the Good Clinical Practice standards. Patients signed written informed consent before enrolment.

### Statistical analysis

We assessed data distribution using the Shapiro–Wilk test. Continuous variables were compared using t-tests in case of Gaussian distribution, while the Mann–Whitney U-test was used for non-normally distributed data. Frequency distribution analysis was performed with Chi-squared or Fisher's exact test. In case of missing data, we used multiple imputation analysis, as previously described in the literature [15]. Statistical analysis was performed using R software (version 4.0.2, R Foundation), considering a p-value <0.05 as statistically significant.

## Results

After evaluating exclusion criteria, 39 patients were included in the final analysis. Main features of the enrolled patients are summarised in table 1, compared to an equally sized random sample of patients suffering from SEA and treated with anti-IL-5/IL-5-receptor from the Southern Italy Network on Severe Asthma Therapy database. Patients with EGPA and SEA were younger than those with SEA (p=0.02), with an earlier diagnosis of severe asthma (p=0.0007) and a more frequent diagnosis of chronic rhinosinusitis with nasal polyps (p=0.03). Moreover, patients with SEA and EGPA tend to have more visits to the emergency department (ED) as well as a higher numbers of hospitalisations (33% *versus* 12.8%, p=0.06). After applying super-response criteria, 71.8% of our patients fulfilled the super-responder definition, without significant anthropometric differences from non-super-responder patients (table 2). Super-responder patients were equally distributed in mepolizumab and benralizumab treatment (50% each). Before starting biologic treatment, all the enrolled patients received an induction therapy with prednisone, with super-responders having higher median OCS prescribed doses (13.7 mg, 95% CI 5–25 mg *versus* 5 mg, 95% CI 2.5–10 mg, p=0.03). According to individual centre preferences and expertise, some patients were also treated with methotrexate (10.7% *versus* 9.1%), azathioprine (28.6% *versus* 18.2%) or rituximab (3.6% *versus* 0), while none of the enrolled patients received cyclosporin or mycophenolate mofetil.

**TABLE 1:** Baseline features of patients with eosinophilic granulomatosis with polyangiitis (EGPA)+severe eosinophilic asthma (SEA) compared to a random sample of patients with SEA from the Southern Italy Network on Severe Asthma Therapy

	**EGPA+SEA**	**SEA**	**p-value**
**Patients**	39	39	
**Age (years)**	51.2±11.4	57.3±11.5	0.02
**Male/female**	35.9/64.1 (14/25)	43.6/56.4 (17/22)	
**BMI (kg·m^−2^)**	24.7±3.6	26.4±4	
**Smoking habits**			
Current smoker	5.1 (2)	7.7 (3)	
Former smoker	23.1 (9)	23.1 (9)	
Nonsmoker	71.8 (28)	69.2 (27)	
**Age at asthma onset (years)**	11.8 (5–19)	23 (14–30)	
**Time from asthma diagnosis (years)**	37.6±11.5	33±13.5	0.0007
**Time from EGPA diagnosis (years)**	10 (4–13.1)	NA	
**Asthma exacerbations**	92.3 (36)	97.4 (38)	
	4 (3–6)	5 (3–7)	
**Access to ED**	41 (16)	25.6 (10)	
**Hospitalisation**	33.3 (13)	12.8 (5)	0.06
**Asthma treatment**			
ICS/LABA	100 (39)	100 (11)	
LAMA	61.5 (24)	61.5 (24)	
SABA	48.7 (19)	59 (23)	
**OCS at baseline**	100 (39)	82.1 (32)	
**OCS dose at baseline (mg)**	10 (5–20)	12.5 (5–25)	
**ACT at baseline**	14.7±5.2	13.6±3.6	
**Comorbidities**			
Eosinophilic pneumonia	15.4 (6)	0	0.02
CRwNP	79.5 (31)	53.8 (21)	0.03
Bronchiectasis	30.8 (12)	23.1 (9)	
Urticaria	7.7 (3)	7.7 (3)	
**Lung function**			
Pre-BD FEV_1_ (%)	76.5±19.9	70.3±23.4	
Pre-BD FEV_1_ (L)	2.25±0.7	1.8±0.9	0.03
Pre-BD FVC (%)	90.7±17.9	87±19.4	
Pre-BD FVC (L)	3.2±0.9	2.8±1.1	
**BEC** **(cells·μL^−1^)**	750 (480–1418)	609 (400–1027)	
**BVAS**	7 (4–9.7)	NA	
**EGPA systemic manifestations**			
Constitutional	20.5 (8)	NA	
Cutaneous	17.9 (7)	NA	
Cardiac	17.9 (7)	NA	
Gastrointestinal	17.9 (7)	NA	
Renal	5.1 (2)	NA	
Peripheral neuropathy	25.6 (10)	NA	
Arthropathy	10.3 (4)	NA	
**Autoantibodies**			
ANA positive	10.3 (4)	NA	
p-ANCA positive	25.6 (10)	NA	
c-ANCA positive	0	NA	
Anti-MPO positive	2.6 (1)	NA	
Anti-PR3 positive	2.6 (1)	NA	
**Immunosuppressant medication**			
Cyclosporin	0	NA	
Mycophenolate mofetil	0	NA	
Methotrexate	10.2 (4)	NA	
Azathioprine	25.6 (10)	NA	
Rituximab	2.6 (1)	NA	

Data are presented as n, mean±sd, % (n) or median (interquartile range), unless otherwise stated. BMI: body mass index; ED: emergency department; ICS: inhaled corticosteroids; LABA: long-acting β-agonists; LAMA: long-acting muscarinic antagonists; SABA: short-acting β-agonists; OCS: oral corticosteroids; ACT: Asthma Control Test; CRwNP: chronic rhinosinusitis with nasal polyps; BD: bronchodilator; FEV_1_: forced expiratory volume in 1 s; FVC: forced vital capacity; BEC: blood eosinophil count; BVAS: Birmingham Vasculitis Activity Score; ANA: anti-nuclear antibodies; ANCA: antineutrophil cytoplasmic antibodies; MPO: myeloperoxidase; PR3: proteinase-3; NA: not applicable.

**TABLE 2:** Baseline features of super-responder patients with eosinophilic granulomatosis with polyangiitis (EGPA)+severe eosinophilic asthma (SEA)

	**EGPA+SEA super-responders**	**EGPA+SEA non-super-responders**	**p-value**
**Patients**	71.8 (28)	28.2 (11)	
**Age (years)**	51.1±11.7	51.5±11	
**Male/female**	39.3/60.7 (11/17)	27.3/72.7 (3/8)	
**BMI (kg·m^−2^)**	24.1±3.5	26.1±3.7	
**Smoking habits**			
Current smoker	7.1 (2)	0	
Former smoker	28.6 (8)	9.1 (1)	
Nonsmoker	64.3 (18)	90.9 (10)	
**Age at asthma onset (years)**	10 (5–18)	13 (5.5–20.7)	
**Time from asthma diagnosis (years)**	36.9±12.3	39.7±9.3	
**Time from EGPA diagnosis (years)**	9 (4–13)	10 (5–17.6)	
**Asthma exacerbations**	96.4 (27)	81.8 (9)	
	4 (3–6)	3 (2–4)	
**Access to ED**	57.1 (16)	0	0.001
**Hospitalisation**	81.2 (13/16)	0	
**Asthma treatment**			
LAMA	67.9 (19)	45.5 (5)	
SABA	60.7 (17)	18.2 (2)	0.02
Mepolizumab	50 (14)	36.4 (4)	
Benralizumab	50 (14)	63.6 (7)	
**OCS dose at baseline (mg)**	13.7 (5–25)	5 (2.5–10)	0.03
**ACT at baseline**	12.8±4	19.5±5	<0.0001
**Comorbidities**			
Eosinophilic pneumonia	21.4 (6)	0	
CRwNP	75 (21)	90.9 (10)	
Bronchiectasis	35.7 (10)	18.2 (2)	
Urticaria	10.7 (3)	0	
**Lung function**			
Pre-BD FEV_1_ (%)	74.7±19.3	81.2±21.6	
Pre-BD FEV_1_ (L)	2.27±0.74	2.2±0.6	
Pre-BD FVC (%)	88.8±17.5	95.8±18.9	
Pre-BD FVC (L)	3.3±1	3.2±0.7	
**BEC (cells·μL^−1^)**	960 (521–1695)	500 (369–890)	0.04
**BVAS**	8 (4–8)	4 (3–12)	
**EGPA manifestations**			
EGPA systemic manifestations	1.6±0.9	2.5±1.1	0.04
Constitutional	25 (7)	9.1 (1)	
Cutaneous	17.9 (5)	18.2 (2)	
Cardiac	25 (7)	0	
Gastrointestinal	25 (7)	0	
Renal	7.1 (2)	0	
Peripheral neuropathy	25 (7)	27.3 (3)	
Arthropathy	3.6 (1)	27.3 (3)	
**Autoantibodies**			
ANA positive	14.3 (4)	0	
p-ANCA positive	17.9 (5)	45.4 (5)	
c-ANCA positive	0	0	
Anti-MPO positive	3.6 (1)	0	
Anti-PR3 positive	3.6 (1)	0	
**Immunosuppressant medication**			
Cyclosporin	0	0	
Mycophenolate mofetil	0	0	
Methotrexate	10.7 (3)	9.1 (1)	
Azathioprine	28.6 (8)	18.2 (2)	
Rituximab	3.7 (1)	0	

Data are presented as % (n), mean±sd, n or median (interquartile range), unless otherwise stated. BMI: body mass index; ED: emergency department; LAMA: long-acting muscarinic antagonists; SABA: short-acting β-agonists; OCS: oral corticosteroids; ACT: Asthma Control Test; CRwNP: chronic rhinosinusitis with nasal polyps; BD: bronchodilator; FEV_1_: forced expiratory volume in 1 s; FVC: forced vital capacity; BEC: blood eosinophil count; BVAS: Birmingham Vasculitis Activity Score; ANA: anti-nuclear antibodies; ANCA: antineutrophil cytoplasmic antibodies; MPO: myeloperoxidase; PR3: proteinase-3.

**FIGURE 1:**
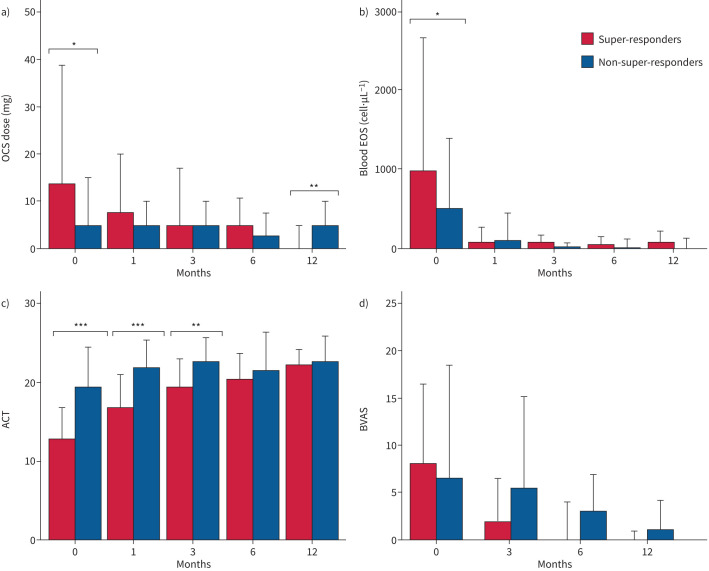
Severe eosinophilic asthma and eosinophilic granulomatosis with polyangiitis features variation in super-responders according to different follow-up times. a) Oral corticosteroid (OCS) dose; b) blood eosinophil (EOS) count; c) Asthma Control Test (ACT); d) Birmingham Vasculitis Activity Score (BVAS). *: p<0.05, **: p<0.01, ***: p<0.001.

**FIGURE 2:**
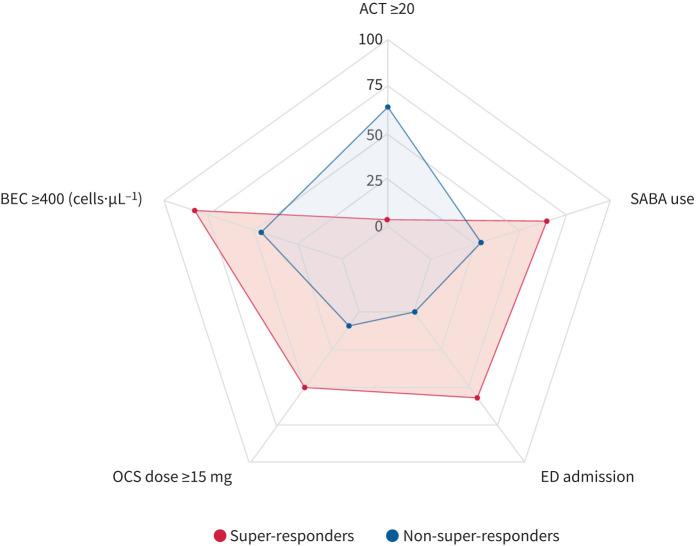
Baseline features of super-responders *versus* non-super-responders. Data are reported in percentages. Blood eosinophil count (BEC) and oral corticosteroid (OCS) dose cut-offs have been chosen for graphical purposes. ACT: Asthma Control Test; SABA: short-acting β-agonist; ED: emergency department.

Super-responder patients suffered more from severe asthma exacerbations at baseline (figure 1), frequently leading to ED access (57.1% *versus* 0%, p=0.001). Moreover, super-responders also had a worse clinical presentation at T0 (figure 2), with lower mean levels of ACT scores (12.8±4 *versus* 19.5±5, p<0.0001) and a frequent use of short-acting β-agonists (SABA) as reliever therapy (60.7% *versus* 18.2%, p=0.02). Finally, median BEC value was higher in super-responder patients (960 cells·mm^−3^, 95% CI 521–1695 cells·mm^−3^ *versus* 500 cells·mm^−3^, 95% CI 369–890 cells·mm^−3^), despite no significant differences in comorbidities (supplementary table E1), BVAS, autoantibodies panel or immunosuppressant treatments.

Table 3 describes main differences in super-responder patients after 12 months of treatment with monoclonal antibodies. All the enrolled patients benefited from biologic treatments, as showed by the reduction of BVAS and EGPA extrapulmonary manifestations at T12. Super-responders did not experience any asthma exacerbation at T12 (0 *versus* 45.5%, p=0.001), in contrast with non-super-responder patients, who also increased access to ED from T0 to T12 (45.5%, p=0.001). Moreover, patients achieving super-response showed a lower annual rate of EGPA relapses compared to non-super-responders (15.4% *versus* 54.5%, p=0.02). As regards OCS administration, super-responder patients significantly reduced OCS use (32.1% *versus* 100%, p<0.0001) and median dose (0 mg, 95% CI 0–5 mg *versus* 5 mg, 95% CI 2.5–5 mg, p=0.004). In parallel, super-responders also had a consistent improvement in ACT scores from T0 to T12 (9.5±3.8 *versus* 3.3±3.4, p<0.0001), with a greater number of patients achieving an ACT increase ≥6 points (92.9% *versus* 18.2%, p<0.0001). In contrast, we found similar lung function values in both our subpopulations, with a higher, but nonsignificant number of super-responders increasing their FEV_1_ >500 mL from baseline (39.3% *versus* 18.2%). Regarding blood eosinophilia, super-responders experienced a significant decrease in BEC compared to non-super-responders (p=0.02), achieving no differences in blood eosinophils values after a 1-year course of biologic treatment.

**TABLE 3:** Features of super-responder patients with eosinophilic granulomatosis with polyangiitis (EGPA)+severe eosinophilic asthma (SEA) after 12 months of biologic treatment

	**EGPA+SEA super-responders**	**EGPA+SEA non-super-responders**	**p-value**
**Total patients**	71.8 (28/39)	28.2 (11/39)	
**Asthma exacerbations**	0	45.5 (5)	0.001
**EGPA relapses**	15.4 (4)	54.5 (6)	0.02
**Access to ED**	0	45.5 (5)	0.001
**OCS use**	32.1 (9)	100 (11)	<0.0001
**OCS dose (mg)**	0 (0–5)	5 (2.5–5)	0.004
**ΔOCS**	10 (5–20)	2.5 (0–7.5)	0.002
**ΔOCS ≥50%**	89.3 (25)	54.5 (6)	0.03
**ACT**	22.5 (20.2–24)	23 (22–25)	
**ACT ≥20**	100 (28)	90.9 (10)	
**ΔACT**	9.5±3.8	3.3±3.4	<0.0001
**ΔACT ≥6**	92.9 (26)	18.2 (2)	<0.0001
**Lung function**			
Pre-BD FEV_1_ (%)	85.2±20.9	89.7±19.9	
Pre-BD FEV_1_ (L)	2.6±0.7	2.4±0.6	
Pre-BD FVC (%)	98.8±20.4	95.9±17.8	
Pre-BD FVC (L)	3.6±1	3.2±0.7	
FEV_1_ ≥500 mL from baseline (%)	39.3	18.2	
**BEC (cells·μL^−1^)**	53 (0–140)	0 (0–101)	
**ΔBEC (cells·μL^−1^)**	835 (453–1675)	368 (318–660)	0.02
**Adverse events**	0	0	
**BVAS**	0 (0–2)	0 (0–1.5)	
**EGPA manifestations**	1±0.9	0.5±0.5	
Constitutional	0	0	
Cutaneous	10.7 (3)	9.1 (1)	
Cardiac	14.3 (4)	0	
Gastrointestinal	14.3 (4)	0	
Renal	0	0	
Peripheral neuropathy	7.1 (2)	9.1 (1)	
Arthropathy	0	0	
**Remission 1**	45.4 (10/22)	25 (2/8)	
**Remission 2**	68.2 (15/22)	75 (6/8)	
**Immunosuppressant medication**			
Cyclosporin	0	0	
Mycophenolate mofetil	0	0	
Methotrexate	3.6 (1)	0	
Azathioprine	10.7 (3)	0	
Rituximab	0	0	

Data are presented as % (n), median (interquartile range) or mean±sd, unless otherwise stated. ED: emergency department; OCS: oral corticosteroids; Δ: change; ACT: Asthma Control Test; BD: bronchodilator; FEV_1_: forced expiratory volume in 1 s; FVC: forced vital capacity; BEC: blood eosinophil count; BVAS: Birmingham Vasculitis Activity Score.

**FIGURE 3:**
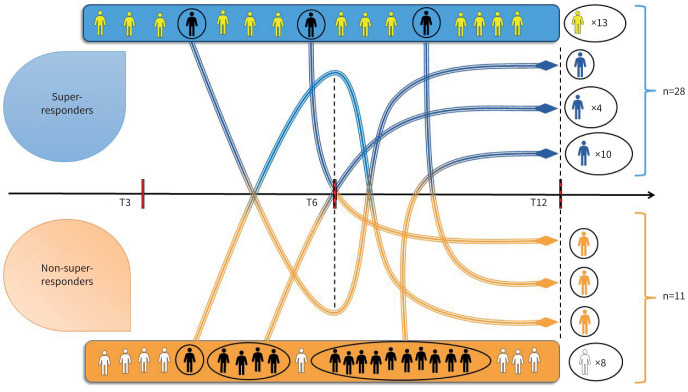
Changes in super-responder and non-super-responder classification during a 12-month follow-up. Yellow and white patients did not change their status over time. T3, T6, T12: 3 months, 6 months and 12 months, respectively, from the start of monoclonal antibody administration.

Considering the improvement in SEA and EGPA clinical features in super-responders, we tested the consistency of super-response definition according to different follow-up time points. As reported in supplementary table E2, the frequencies of super-responders without exacerbations and without a current OCS treatment reached a significant difference only at T12 (p=0.017 and p=0.0008, respectively). Frequencies of improvement in ACT scores ≥6 points from baseline arise in super-responders starting from T3 (p=0.08), becoming significant at T6 (p=0.01) and T12 (p<0.0001). Finally, only the frequency of ACT ≥20 was lower in super-responders at T1 (p=0.03), without any further difference from T3 to T12. Super-response definition lacks enough sensitivity when applied before 12 months from the start of biologic therapy (figure 3, supplementary table E3), since 23 patients were misclassified before the T12 visit (15 as non-super-responders and eight as super-responders).

Finally, we evaluated EGPA remission rate according to the aforementioned criteria. Since nine patients lacked BVAS assessment at T0 or T1, we excluded them from EGPA remission assessment. In the remaining cohort, “remission 1” and “remission 2” were achieved in 45.4% and 68.2% of super-responders, respectively, without significant differences compared to non-super-responders.

## Discussion

To the best of our knowledge, this is the first study assessing whether super-response criteria could be applicable to patients with SEA with comorbid EGPA after a 12-month course of treatment with mepolizumab or benralizumab. The aim of our study was not to apply a severe asthma super-responder definition to patients with EGPA, but to explore its applicability in patients suffering from SEA and other eosinophilic-driven comorbidities. Indeed, super-response definitions have been proposed in patients with severe asthma only [8], while the impact of comorbidities on severe asthma super-responder criteria has still to be established [5].

Our results show that >70% of the enrolled patients could be classified as super-responders, confirming the dramatic impact of monoclonal antibodies on SEA and EGPA management [16–19]. Moreover, these results have also been achieved in patients treated with mepolizumab 100 mg, which is a third of the dose licensed for EGPA. Nevertheless, our data are in line with those previously published considering mepolizumab 100 mg in patients with relapsing-refractory EGPA [20–22]. Despite the lack of a unique definition of super-responders, the characterisation of super-responder populations to individual therapy may help physicians better select a tailored treatment able to achieve patients’ clinical remission [23]. However, several considerations arise when dealing with patients suffering from SEA and comorbid EPGA. Severe asthma super-response definition using the lack of OCS as a major criterion could be troublesome to achieve in these patients, since systemic corticosteroids are still frequently used as EGPA maintenance treatment. In our cohort, 32.1% of super-responders were still treated with OCS even after 12 months of biologic treatment, a slightly lower percentage compared to the MIRRA trial [13]. However, real-life experiences using mepolizumab and benralizumab in patients with EGPA reported high variability in OCS withdrawal during follow-up [3, 18, 19, 24]. These data confirm how challenging it could be to define super-responders only using OCS cessation. An alternative criterion to classify super-responders could rely on OCS withdrawal during follow-up, considering a reduction of ≥50% of the baseline administered dose as a super-response to treatment [2, 7, 25]. Among our enrolled patients, three (27.3%) non-super-responders could be reclassified according to this definition, confirming a possible role for OCS reduction as a major super-responder criterion when SEA and EGPA coexist.

As regards super-responder features, our results suggest a more severe clinical baseline SEA phenotype compared to non-super-responder patients. In fact, super-responders showed worse symptoms, higher rates of exacerbations leading to ED admission, greater levels of BEC, more frequent use of SABA as a reliever and higher OCS doses used to control SEA and EGPA. In patients with SEA only, data reporting baseline symptoms in super-responder patients are discordant. While some evidence suggests that lower baseline ACQ scores could foreshadow a greater response to biologics [5, 6], other studies reported different results [26, 27]. A possible explanation could be related to higher T0 BEC in super-responders, which could explain their worse baseline clinical status. The fast drop in BEC obtained with monoclonal antibodies targeting the IL-5 axis can be reasonably considered crucial for SEA super-response, since three main aspects of super-responder definition were positively affected after biologic administration (exacerbations, OCS dose, ACT).

A crucial point of our results concerns the relationship between super-responders and EGPA remission. Regardless of the criteria used, we did not find any significant difference in the remission rate of super-responder *versus* non-super-responder patients. Moreover, apart from a higher baseline level of BEC and a lower number of systemic EGPA manifestations in super-responder patients, there were no other relevant differences according to treatment super-response. The analysis of these results led us to some interesting considerations. In our opinion, diagnostic criteria to define super-responders seem to be too far from those used in the definition of EGPA remission. In fact, super-responder criteria frequently include the lack of SEA exacerbations and functional improvement, which are not included in the definition of EGPA remission. Moreover, as stated previously, it could be hard to cease OCS in patients with EGPA. Consequently, some patients with great responses to biologic treatment could be misclassified due to residual OCS use. In our cohort, 67.9% of super-responders are free from OCS administration at T12, but 89.3% had an OCS dose reduction ≥50% from baseline, with a significant difference compared to non-super-responders (p=0.03; table 3).

Another important aspect is related to the frequency at which major and minor super-responder criteria are fulfilled. Among super-response features, only ACT improvement can be reliably assessed earlier during the 1-year follow-up, since the difference in the absence of exacerbations and in the OCS use can be achieved only at T12 in super-responders. These results suggest that the main differences between super-responders and non-super-responders on specific outcome variables (exacerbations, OCS) can only be assessed after 12 months of continuous treatment with monoclonal antibodies. Differently, respiratory symptoms improve earlier and faster in super-responders, despite a worse clinical presentation at baseline. Kavanagh *et al.* [6], in their assessment of super-response to mepolizumab administration in patients with SEA, concluded that it seems possible to define super-responders even after 24 weeks of treatment, with good levels of sensitivity and specificity compared to a 12-month definition. Our results are in contrast with this statement, especially for the lower level of sensitivity for super-responder detection reached at T6 (supplementary table E3). As shown in figure 3, a high percentage of patients switched from one category to the other, especially from non-super-responder to super-responder. These changes are more frequent, passing from T6 to T12, while only 46.4% of patients achieve super-responder criteria in every follow-up time. A possible reason for such difference could be related to baseline population features. Kavanagh *et al.* [6] performed their analysis in patients with SEA only, while we focused on patients with SEA and EGPA. The presence of EGPA could have slowed some patients’ response to monoclonal antibodies, causing a delay in the switch from non-super-responder to super-responder. Moreover, OCS-weaning practices have a pivotal impact on super-responder definition, especially during the first phase of the follow-up.

Our study has several limitations. First, the lack of a control group could have influenced our conclusions, albeit a common bias of real-life studies. Second, super-response criteria still need to be standardised; therefore, our super-responder classification could be considered subjective. However, we used a wide subset of super-responder criteria, investigating every central aspect of SEA treatment response. Consequently, our results could reflect a real super-response pattern to biologic treatment for patients suffering from SEA and EGPA. Finally, we only assessed super-responder after 12 months, which could have been useful to verify the consistency of super-response definition in a 1-year follow-up. Notwithstanding these limitations, the major strengths of the study are the multicentre nature that allows for the generalisability of the results and the simultaneous characterisation of the super-responder phenomenon with anti-IL-5 therapies for eosinophilic disease that may drive clinicians in choosing between targeting therapies in patients with SEA and EGPA.

In conclusion, we reported real-life evidence of the efficacy of mepolizumab and benralizumab treatment in patients with EGPA and SEA achieving fulfilling super-response definition. Despite a worse baseline clinical scenario, super-responders have greater response to biologic treatment in terms of reduction in severe exacerbations, BEC decrease, symptoms management and OCS administration.

## Supplementary material

10.1183/23120541.00419-2023.Supp1 **Please note:** supplementary material is not edited by the Editorial Office, and is uploaded as it has been supplied by the author.
Supplementary material 00419-2023.SUPPLEMENT
