# Supplementary material for: Real-world characteristics of “super-responders” to mepolizumab and benralizumab in severe eosinophilic asthma and eosinophilic granulomatosis with polyangiitis
Source: ERJ Open Res. 2023 Oct 30;9(5):00419-2023. doi: 10.1183/23120541.00419-2023 (PMC10613971; doi:10.1183/23120541.00419-2023)

**eTable 1**. Comorbidities of SR patients with EGPA+SEA

| | **EGPA+SEA SR** | **EGPA+SEA Non-SR** |
|---|---|---|
| **Total patients (%, n)** | 71.8 (28/39) | 28.2 (11/39) |
| Total comorbidities (mean, SD) | 3.2 ± 1.9 | 2.2 ± 1.5 |
| **Comorbidities** | | |
| ○ Eosinophilic pneumonia | 21.4 (6) | 0 |
| ○ CRwNP | 75 (21) | 90.9 (10) |
| ○ Bronchiectasis | 35.7 (10) | 18.2 (2) |
| ○ Urticaria | 10.7 (3) | 0 |
| ○ Vocal cord dysfunction | 3.6 (1) | 0 |
| ○ GORD | 32.1 (9) | 36.4 (4) |
| ○ OSAS | 14.3 (4) | 0 |
| ○ Osteoporosis | 35.7 (10) | 36.4 (4) |
| ○ Anxious-depressive syndrome | 39.3 (11) | 18.2 (2) |

SR, Super-responders; SD, Standard Deviation; CRwNP, Chronic Rhinosinusitis with Nasal Polyps; GORD, Gastroesophageal Reflux Disease; OSAS, Obstructive Sleep Apnea Syndrome

**eTable 2**. Comparison between major and minor SR criteria according to different follow up times. Fisher's tests refer to the same follow up visit.

| | EGPA+SEA SR | | | | EGPA+SEA Non-SR | | | |
|---|---|---|---|---|---|---|---|---|
| | T1 | T3 | T6 | T12 | T1 | T3 | T6 | T12 |
| **Major criteria (%, n)** | | | | | | | | |
| • No OCS | 10.7 (3) | 14.3 (4) | 35.7 (10) | 39.3 (11)* | 0 | 9.1 (1) | 9.1 (1) | 0 |
| • No exacerbations | 82.1 (23) | 75 (21) | 82.1 (23) | 100 (28)*** | 90.9 (10) | 81.8 (9) | 90.9 (10) | 54.5 (6) |
| • ΔACT ≥ 6 | 28.6 (8) | 60.7 (17) | 67.9 (19)** | 92.9 (26)*** | 18.2 (2) | 27.3 (3) | 18.2 (2) | 18.2 (2) |
| **Minor criteria (%, n)** | | | | | | | | |
| • ACT ≥ 20 | 32.1 (9)* | 67.9 (19) | 71.4 (20) | 100 (28) | 72.7 (8) | 90.9 (10) | 72.7 (8) | 90.9 (10) |
| • Exacerbations reduction ≥ 75% | 78.6 (22) | 78.6 (22) | 92.9 (26) | 100 (28) | 81.8 (9) | 72.7 (8) | 81.8 (9) | 81.8 (9) |
| • ΔFEV1 ≥ 500 ml | 14.3 (4) | 28.6 (8) | 28.6 (8) | 39.3 (11) | 18.2 (2) | 27.3 (3) | 18.2 (2) | 18.2 (2) |

* P<0.05; **P<0.01; *** P<0.001

SR, Super-responders; OCS, Oral Corticosteroids; ACT, Asthma Control Test

**eTable 3**. Sensitivity, specificity, false positives and negatives for SR definition according to follow up times

| | T1 | T3 | T6 |
|---|---|---|---|
| False Positives (n) | 3 | 2 | 2 |
| False Negatives (n) | 24 | 14 | 11 |
| Sensitivity (%, 95% CI) | 14.3 (5.7 – 31.5) | 50 (32.6 – 67.4) | 60.7 (42.4 – 76.4) |
| Specificity (%, 95% CI) | 72.7 (43.4 – 90.3) | 81.8 (52.3- 94.9) | 81.8 (52.3- 94.9) |

CI, Confidence Interval

**eFigure 1**. Flow diagram for patients screening and enrollment from "Southern Italy Network on Severe Asthma Therapy"

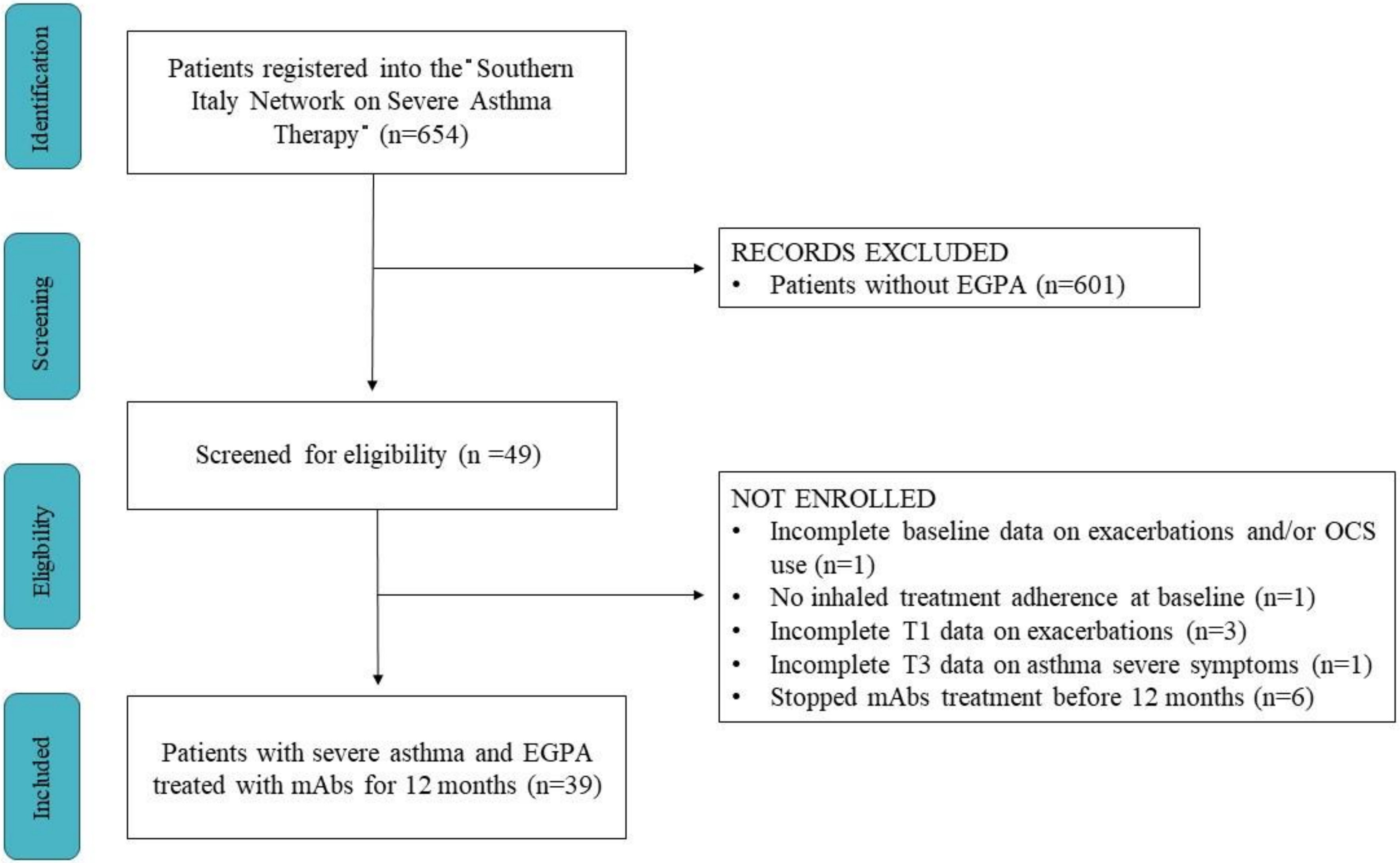

**eFigure 2**. ACT and OCS follow-up variations in patients treated with Mepolizumab, Benralizumab and in the overall population.

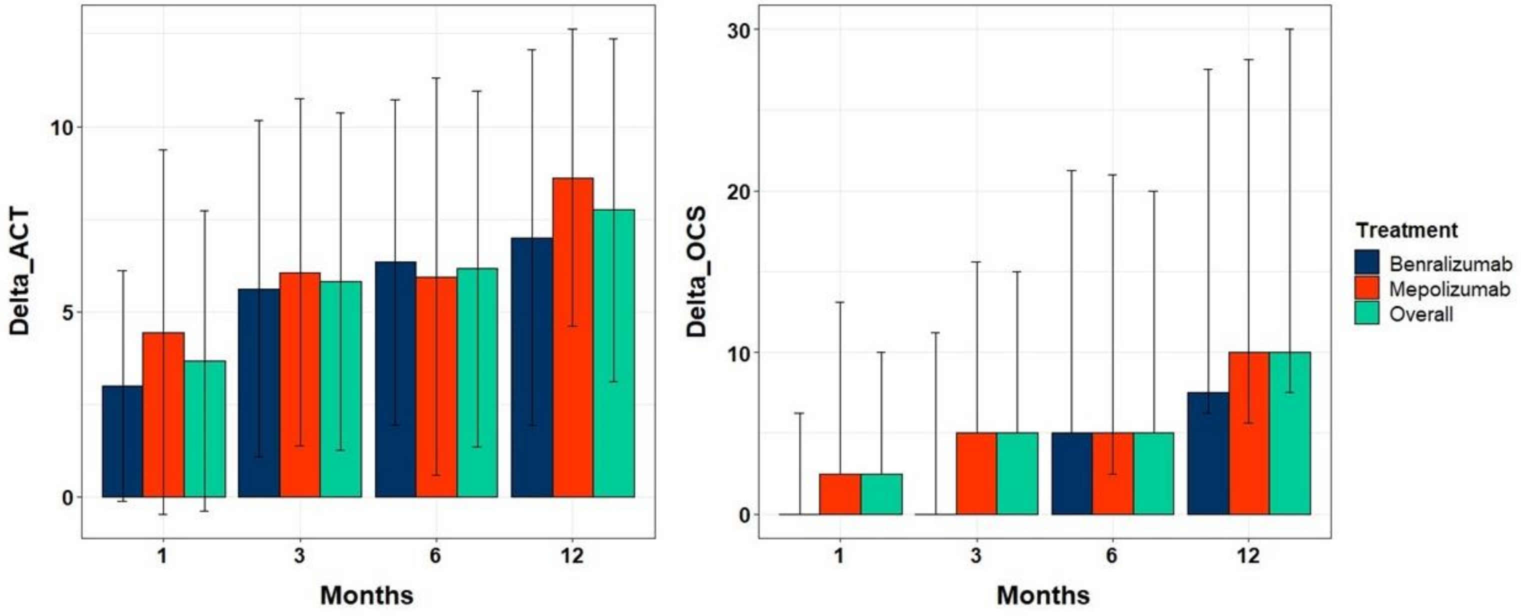

**eFigure 3**. Blood Eosinophils drop and super-response assessment in patients treated with Mepolizumab, Benralizumab and in the overall population

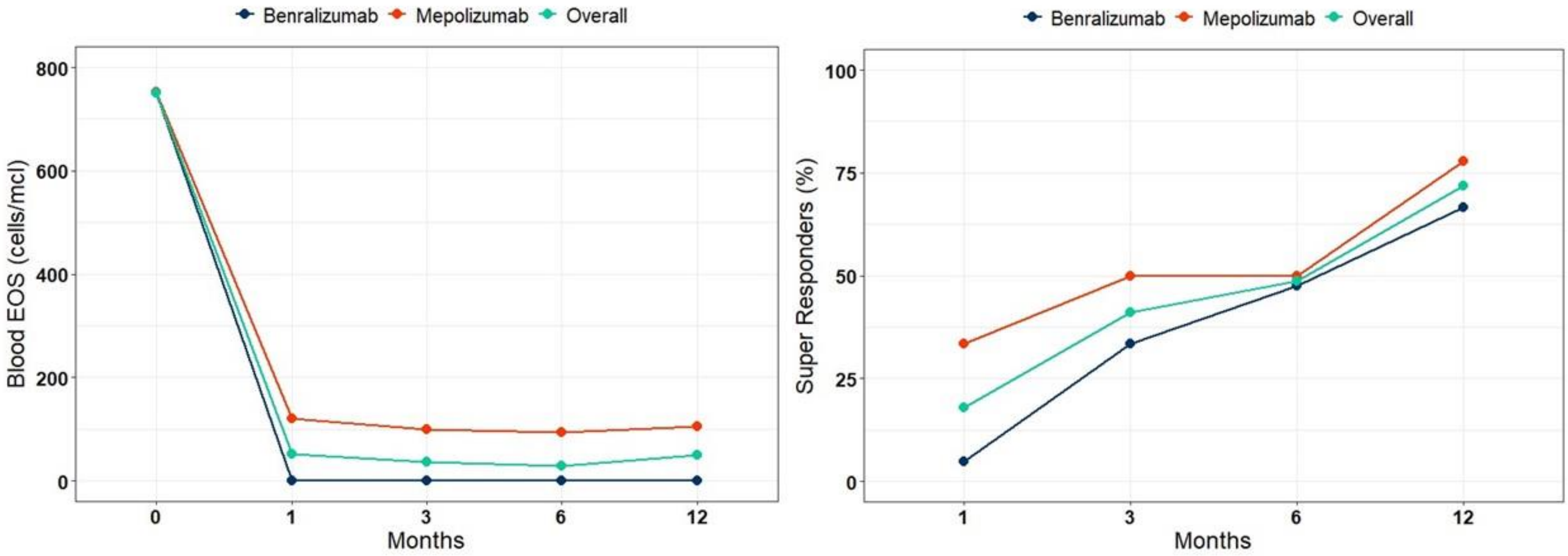

Supplement: Supplementary file 1 [file 00419-2023.SUPPLEMENT.pdf]
